# Do women’s perspectives of quality of care during childbirth match with those of providers? A qualitative study in Uttar Pradesh, India

**DOI:** 10.1080/16549716.2018.1527971

**Published:** 2018-10-08

**Authors:** Sanghita Bhattacharyya, Aradhana Srivastava, Malvika Saxena, Mousumi Gogoi, Pravesh Dwivedi, Katie Giessler

**Affiliations:** a Research Department, Public Health Foundation of India, NCR Delhi, India; b Global Health Sciences, University of California, San Francisco, CA, USA

**Keywords:** Quality of care, public health facilities, maternal health, delivery care, person centred care, qualitative data, India

## Abstract

**Background**: Persistently high maternal mortality levels are a concern in developing countries. In India, monetary incentive schemes have increased institutional delivery rates appreciably, but have not been equally successful in reducing maternal mortality. Maternal outcomes are affected by quality of obstetric care and socio-cultural norms. In this light there is need to examine the quality of care provided to women delivering in institutions.

**Objective**: This study aimed to examine pregnant women’s expectations of high-quality care in public health facilities in Uttar Pradesh, India, and to contrast this with provider’s perceptions of the same, as well as the barriers that limit their ability to provide high-quality care.

**Methods**: A qualitative descriptive analysis was conducted on data from two studies – focus group discussions with rural women in their last trimester of pregnancy (conducted in 2014) to understand women’s experience and satisfaction with maternal care services, and in-depth interviews with care providers (conducted in 2016–17) to understand provision of person-centred care. Provider perspectives were matched with themes of women’s perspectives on quality of childbirth care in facilities.

**Results**: Major themes of care prioritised by women included availability of doctors at the facility; availability of medicines; food; ambulance services; maintenance of cleanliness and hygiene; privacy; good and safe delivery with no complications; client-provider interaction; financial cost of care. Many women also voiced no expectation of care, indicating disillusionment from the existing system. Providers concurred with women on all themes of care except availability of doctors, as they felt that trained nurses were proficient in conducting deliveries.

**Conclusions**: This study shows that women have clear expectations of quality care from facilities where they go to deliver. Understanding their expectations and matching them with providers’ perspectives of care is critical for efforts to improve the quality of care and thereby impact maternal outcomes.

## Background

Despite concentrated global efforts to decrease the incidence of maternal mortality in low-resource settings, the maternal mortality ratio (MMR) remains unacceptably high. In 2015, the MMR for low and middle-income countries (LMICs) persisted at 239 deaths per 100,000 live births, as compared to just 12 deaths per 100,000 live births in developed countries [1]. With a population of more than one billion people, India has been significantly impacted by high maternal mortality rates. In response, a myriad of strategies were implemented to reduce India’s MMR, most notably the National Rural Health Mission (NRHM). In 2005, the Indian National Government launched the NRHM to improve access to health services for rural populations of India, and to reduce maternal and infant mortality rates [2]. Under the NRHM, there was a notable push for rural women to deliver in health facilities with skilled birth attendants. Programs such as the Janani Suraksha Yojana (JSY) program were put into place to monetarily incentivise women to do so [2,3]. As a result, institutional delivery rates in India have increased from 38.7% in 2005 to almost 80% in 2015–2016 [3,4]. Although JSY has been successful in facilitating a marked increase in institutional delivery, this increase has not been associated with a reduction in MMR as initially anticipated [3]. This could be attributed to gaps in the quality of care received by women at the facilities.

The World Health Organisation (WHO) states that obstetric care should be: safe; effective; timely; efficient, equitable; and people-centred [5].

Provision of high-quality obstetric care depends on a wide range of structural inputs and effective processes being performed. But deliveries happen in the context of social-cultural norms, so it is important to understand the expectations and experiences of both women and providers, as they can impact maternal health outcomes. In spite of high clinical quality, if women are mistreated, demanded money or demeaned during the process of delivery, they will avoid and delay utilisation of medical care as much as possible [6,7].

A 2016 synthesis of literature from LMICs indicated that interpersonal behaviour was the most widely reported determinant of satisfaction with maternity care by women, along with availability of drugs and equipment on the structural side [8]. Consumers of maternity services are becoming increasingly aware with regard to expectations of quality, with some women in LMICs even bypassing more easily accessible, local primary care clinics in search of higher-quality care at secondary and tertiary level facilities [9]. Perceptions surrounding the quality of care that will be received in a facility impact a woman’s initial decision to deliver in a facility, in addition to whether she will access institutional delivery for subsequent births [6,7,10]. Although women in LMICs have reported that high-quality interpersonal interactions are directly related to satisfaction with care, research suggests that providers may consider elements such as respect, privacy, dignity and information-sharing to be less critical components of care that require immediate attention [11].

There remains a dearth of literature that specifically compares patient and provider perspectives on what constitutes high-quality maternity care. Without a shared definition of quality, interventions that target improved quality may be lacking for either the patient, or the provider, or for both. In this paper, we aim to examine pregnant women’s expectations of high quality care in public health facilities in Uttar Pradesh, India, and to contrast this with provider’s perceptions of the same, as well as the barriers that limit their ability to provide high-quality care. Uttar Pradesh (UP), the most populous state in India, accounts for more than 20% of overall maternal deaths in India [12]. Normalisation of mistreatment in the facility context may lead to low expectations among women to receive high quality care [6]. Comprehensive understanding of the challenges that exist for health care practitioners to provide high-quality care across structural, staffing, supply-chain and procedural processes has the potential to inform targeted interventions to improve the overall provision and experience of care for patients, presumably leading to decreases in death and disability from maternal complications in UP.

## Methods

The paper is a qualitative descriptive analysis based on data from two studies conducted in the same geographical stetting. Focus group discussions (FGDs) with rural women in their last trimester of pregnancy were conducted between April and May 2014 as part of a study to understand women’s experience and level of satisfaction with maternal care services. In a subsequent study on person-centred maternal care, in-depth interviews (IDIs) with care providers were carried out between October 2016 and February 2017.

## Study setting

The studies were conducted in two districts in the state of Uttar Pradesh (UP) in Northern India. UP is a high-priority state, as it is among the bottom-line performers in terms of health indicators; with a high infant mortality rate (46 per 1000 live births) [13] and maternal mortality ratio (285 per 100,000 live births) [14] as compared to national rates. The two study districts were selected based on their performance in maternal and child health indicators. District - A had relatively better MCH indices with 76% institutional deliveries (69% in district-B) and 79% deliveries assisted by skilled personnel, as compared to district – B (71%) [15,16]. Both the study districts show comparative health system characteristics having similar number of functional CHCs (District A – 14; B – 10) and PHCs (District A – 53, B – 51) [17]. There is no shortfall of Staff Nurses in position in the two study districts, though at the state level there is a 2% shortfall [18,19]. A range of primary- and secondary-level facilities were included in an attempt to understand provider and system-level challenges specific to both levels and ensure heterogeneity in the data for wider implications.

## Study instruments

Semi-structured interview guides were used in the data collection. Open-ended questions were supported with probes where necessary. The IDI and FGD guides were developed based on the Hulton framework for quality of care in maternal health services [20]. The framework is specifically developed to asses quality of maternity care within institutional contexts and includes themes like access and referral, human and physical resource, respect and dignity, privacy, cognitive support, emotional support and cost of care. The guides were also informed by our previous research on quality of maternal healthcare services in India [21]. The quality themes covered in the guides included access and connectivity to the facility, infrastructure, provider availability, promptness of care; appropriate medical care; faith in provider’s competency, emotional support, privacy; cleanliness and hygiene; interpersonal behaviour, and information sharing with women on their condition. The instruments were translated into the local language of the study area (Hindi), back-translated and pretested before they were finalised.

## Sampling and data collection

FDG participants were women in the last trimester of pregnancy and were identified using a community health worker list. The research team approached every third eligible woman from the list, explained the purpose of the study, and sought her verbal consent for participation. Five FGDs were conducted with seven to eight participants each; the total respondent sample for the FGDs was 36 women. It was ensured that the groups included both primi and multigravida women for variation of responses on delivery care expectation and experiences.

For the IDIs, three cadres of providers were interviewed in each facility – the Medical Officer In-Charge (MOIC), one Lady Medical Officer (LMO), and one Staff Nurse (SN) directly involved in providing maternity care. A total of 27 IDIs were conducted across nine public health facilities ranging from primary health center (PHC) to a secondary-level community health center/first referral unit (CHC/FRU).

Two people comprised the research team, who were experienced qualitative researchers and knew the local dialect of the study area. The senior researcher in the team led the interview and was supported by the junior researcher. The FGDs were conducted in a common but secluded area of the village, and the research team ensured that no provider or community health worker was present during the discussion. At the beginning of the FGD, the facilitators explained the general topics and encouraged the participants to express their ideas freely. Care was taken to ensure that all themes of care were covered in the discussion. Participants were also encouraged to discuss any other theme that emerged. On an average the duration of each FGD was 1.5 hours. Two researchers with experience in qualitative research and knowledge of local dialect of the area conducted the FGDs; one moderated the discussion while the other took notes.

IDIs were conducted at the health facilities themselves, in a secluded space with no disturbance. Approximate duration of each IDI was 30–45 minutes. At the end of each IDI or FGD, the researchers completed a section on notes and observations that included perceptions as to whether respondents answered questions freely, ease of the interview, and any other relevant information. A debriefing was held at the end of each day, to reflect upon the interviews conducted and accordingly improve the subsequent interviews. Interviews and FGDs were audio-recorded after due informed consent from the participants. An independent researcher transcribed the interviews into the local dialect (Hindi). The transcripts were then translated into English for ease of coding and analysis.

## Data analysis

The analysis was based on the thematic approach. Initially, ‘a priori’ codes were identified from the topic guides, to which emerging themes from the transcripts were added. Two levels of thematic codes were developed and applied to the data. Initially one researcher listed *a priori* themes based on the FGDs that focused on women’s perspectives on quality of childbirth care in facilities. The provider perspectives on the corresponding themes were then identified and added. Following this, two researchers from the study team jointly identified a set of emerging themes based on synthesis and cross-comparison of data from different groups of respondents in the two study districts. Atlas TI software was used to systematically review and code the data. All themes were first broadly categorised as structure and process of care as per the Donabedian framework and further sub categorisation of the themes was based on Hulton’s framework [20,22–24]. The final analysis identified 10 major themes, which women in three or more FGDs prioritised for childbirth in health facilities. These themes were matched with providers’ perspective and challenges associated, if any, to provide such care.

## Ethical approval

This study obtained ethical approval from the University of California, San Francisco (153,312) and the Institutional Ethics Committee of the Public Health Foundation of India (TRC-IEC-276/15; TRC-IEC-187/13). Written permission from the State Mission Director, National Health Mission and the Chief Medical Officers of the two districts was obtained to undertake the study in the selected facilities before starting data collection. Verbal consent was obtained from women who participated in the study. Written consent was obtained from providers who participated in interviews. Anonymity of identity and confidentiality of information was assured to all participants during analysis by masking participant details and village/facility identities.

## Profile of respondents

Most women who participated in the FGDs were 23–26 years old, illiterate, Hindu and of poorer socio-economic status. Husbands of most women worked as casual labourers in the non-agricultural sector. Most respondents had completed three ANC visits in government facilities. Most women were pregnant with their second child, and had previously delivered in a government health facility (Supplemental online material – Appendix 1). All providers interviewed were actively involved in the process of maternity care. The majority had more than five years’ experience in the medical profession and had been in their present position for more than three years. The MOIC and LMOs held Bachelor’s of Medicine, Bachelor of Surgery (MBBS) degrees and the nurses had a Diploma in General Nursing and Midwifery (Supplemental online material – Appendix 2).

## Results

Results are arranged by the themes of expectation of care prioritised by women during childbirth in health facilities. Their perspective is followed by the providers’ perspective on those themes along with the usual practice and the challenges they face in delivering such care.

Themes that emerged as important to both women and providers were common, though often eliciting contrasting views. Themes are organised by first the structural elements of what women felt that a facility should essentially have to provide delivery services – doctors, medicines, food, ambulances, cleanliness. These are followed by elements of process of care prioritised by women once admitted for delivery, reflecting how women want to be treated – ensuring privacy, good care and safe delivery, respectful behaviour and adequate information sharing. Following this we discussed the financial burden imposed by costs incurred on medicines and tests from outside, and informal payments. Last, we have summarised responses of several women voicing their disillusionment and lack of expectation of care at facilities, which is also reflective of the health system’s non-responsiveness to women’s needs.

## Major themes of care

### Structural basics in a facility: doctors, medicines, food, ambulances and cleanliness

#### Doctors available at the facility and examining women: women complain of their absence; providers cite duty hours, shortage of female doctors

Perhaps the most pressing requirement for women reaching a facility was to find a doctor present there, who could examine them. Women expressed that while they would be more satisfied if they were seen by a doctor but often their availability in the facility was uncertain. Women narrated instances either from their own or other’s experiences when doctors were not often available at the facility or would not see patients unless there was a complication.

*‘[The doctors] come to see the patient in case of complication only; yes, they are available in the facility if we want to meet them but they usually don’t come to see normal deliveries.’* (FGD, Women).

The providers attributed women’s perception of doctors not being available in facilities to their lack of knowledge of doctor’s working hours. Doctors usually attended outpatients and were called to attend deliveries only if the delivery load was high or if there was a complication.

*‘Actually, patients feel that doctors don’t stay here because they don’t know what the duty hours are for doctors as deliveries happen more at night time. There is no permanent doctor [residing at facility]*.’ (IDI, LMO).

Doctors also highlighted a shortage of female doctors which affected the availability of doctors to examine women, who preferred female providers for maternity care. But while there were female doctors posted at facilities, their attendance in the facility was affected by security concerns and lack of amenities.

*‘Doctor’s availability is a problem. Male doctors can’t do deliveries – women are shy and don’t let them do so. But female staff is not enough in number and hence we are forced to get contractual staff.’* (IDI, MOIC).

### Availability of required medicines free of cost at the facility and provision of other life saving supplies: women complain of non-availability, providers blame shortages in supply

Medicines required for delivery are supposed to be available free of cost to women at public health facilities. However, women were concerned as these were often not available and women were asked to procure them from outside.

‘*And they should give all prescribed medicines free of cost or reimburse our money if we buy them from outside. Generally the trend is that most of the prescribed medicines are not available in the hospital and one has to get them from outside*.’ (FGD, Women).

Providers realised that this was a problem for patients but explained that patients had to be asked to get medicines from outside only when medicines were out of stock. Their replenishment generally takes time as medicines are procured at the state level and then disbursed to facilities. This was frustrating for the providers too.

Some providers said that the patients themselves request medicines that are often not available at the facility, but these are prescribed only if there is a need and not otherwise.

*‘Patients who come here are mostly sensible, like if a medicine is not available here they ask us to prescribe it so they can get it from outside. We only prescribe medicines from outside if there is a need; otherwise, we tell them that this medicine is sufficient for them.’* (IDI, LMO).

Most of the providers pointed to an inability to manage complications at the facility level, owing to lack of requisite infrastructure or supplies, leading to referral of complicated cases to a higher-level facility as opposed to managing the complication at their own facility.

*‘We always ask to mark out high risk pregnancies beforehand and do not get them delivered here and tell them well in advance that this service is not available in this facility as in our facility there is no provision of blood transfusion; so if there is an anaemic patient we refer them so it’s better if a child is delivered and blood transfusion can be done simultaneously in other facilities.’* (IDI, MOIC).

### Availability of good-quality food at the facility for women and attendants: common concern for both women and providers

Availability of food was considered important by women as it affected their length of stay at the facility. Food was required not only for the women but also their attendants, who accompany them through the duration of their stay at the facility.

‘*Definitely we should get food in the hospital otherwise we have to carry our own food as we have to stay there for days together. The food should be of good quality*.’ (FGD, Women).

Providers also considered provision of food as one of the essential requirements for delivering women. Realising the difficulty families faced in arranging food for the attendants who stay back with women after delivery, some providers suggested that the Government should provide for food for the attendant as well under the JSSK scheme.

MOICs also expressed their concern over the quantity and quality of food supplied at the facility for women. Contracts to meal suppliers were issued by the District office and the MOICs had no authority over them, which constrained their ability to monitor the quality of food supplied to the facilities.

‘*I also want that the external meal supplier under the JSSK program should instead be converted to a system of recruiting only local suppliers so that the quality of the food might be monitored and improved. Even though I am the head of this facility I have no control over the external supplier. I cannot ask him to improve quality of food he supplies in my facility, as this contractor is monitored and controlled by the District unit*.’ (IDI, MOIC).

### Availability of well-equipped ambulance services at all times to transport women to the facility in emergency, or for referrals: emphasised by both women and providers

Another key requirement for a facility is the availability of ambulances for timely transport of women to the facility during emergencies and for timely referrals. Women expressed the importance of having such a vehicle to reach the facility during emergency. While earlier there was no such service, they were appreciative of the recently launched ambulance services that had improved access to facilities for delivery.

*‘There was no ambulance facility even for a patient with serious condition or during medical emergency. Now, the ambulance arrives immediately upon dialing 102.’* (FGD, Women).

Providers also expressed satisfaction with the availability of ambulances but pointed out difficulty in ambulance availability at night as the drivers were reluctant to drive at night and would sometimes refuse to pick up women in need of services. Providers suggested improving ambulance services to serve as mobile medical or delivery units, to reach safe delivery services to women in difficult to access areas.

‘*Access is the biggest challenge in rural areas. If there is a pregnant woman in a village who is having trouble and she will not be able to come here, how you will manage it there? How do we reach villages which are 15 to 20 km away? You can send the ambulance, but not the facilities, and till they come here, there might be delay. There should be a mobile team with trained workers. Vehicles are available, no doubt, but not with trained workers. According to me, if on 102 [ambulance], they should be trained in labour and delivery, and females should be used, because if they are men the patient will not let her delivery be conducted by them. There should be a mobile labour room with an expert staff nurse*.’ (IDI, MOIC).

### Cleanliness in the facility with good hygiene and sanitation: common concern for both women and providers due to inadequate cleaning staff

Women in most FGDs cited the need for cleanliness in the facilities, which included both overall cleanliness, and the need for clean toilets and bed sheets.

*‘There was no cleanliness back then, but this time I expect that it should be there, bed sheets should be clean…Why would the mother and baby stay in an unclean environment as it gives rise to diseases?’* (FGD, Women).

Providers also expressed similar views on maintaining hygiene and cleanliness, but felt handicapped in ensuring that due to lack of adequate cleaning staff at facilities.

*‘A hospital should be a place free from any infection but appropriate level of hygiene and sanitation is not being maintained, clean drinking water and toilets are the two basic minimum facilities that should be made available at all times to staff as well the patients. Basically, the working conditions are untidy as we have [only a] single sweeper in the whole hospital’* (IDI, MOIC).

### Process of care centred on women’s needs: privacy, good care, respectful and adequate communication

#### Ensuring women’s privacy: common concerns for women and providers; measures include use of curtains, screens and no entry to outsiders

To ensure privacy, women in most FGDs cited the need for screens between beds and delivery tables.

*‘Yes, definitely there should be a partition between two delivery tables. Yes, it would be much better if they provide such facility. But at the time of emergency, these things don’t bother [us] that much.’* (FGD, Women).

Providers also prioritised ensuring privacy in childbirth services. They highlighted the use of curtains and separators, though also pointed out that lack of space often restricted the use of the latter. Half of the providers emphasised on restricted entry of males in the examination and labour rooms as an important privacy measure.

*‘There are curtains in the labor room and I have also made the window glasses of labor room opaque to maintain more privacy. Males are prohibited from entering the labor room…and only one woman can be in there during checkup.’* (IDI, MOIC).

#### ‘Good’ care: prompt, respectful care, effective medicines, safe delivery with no complications for women; appropriate clinical care and timely referrals for providers

All women desired the facility staff to provide ‘good’ care. However, ‘good’ care had multiple connotations, ranging from respectful care to clinically appropriate care, and also included structural elements like availability of staff, medicines, electricity and cleanliness. ‘Good’ care also included prompt care and constant monitoring, administering pain relief when required, and availability of good-quality medicines free of cost. Women also associated good care with safe delivery without any complications, leading to a healthy mother and newborn. Women stated that the main reason for preferring hospital over home delivery was the likelihood of timely management of any complication.

*‘During delivery complications may arise any moment; once you are in the hospital it is easy to manage complications but if you are delivering at home and complications arise then it will be difficult to reach the hospital and this will delay management of complications.’* (FGD, Women).

The need for good and safe delivery care was also echoed by half of the providers; who expressed that complications should be recognised on time, ensuring that both the mother and baby are healthy.

*‘Last year or two years back, I referred a patient. She was multigravida and all the conditions were there for normal delivery. She was admitted since morning and had no issues; her only issue was her BP which was slightly high, 140/90. There were no such difficulties, so I thought maybe BP was rising due to pain. At night, around 2–2:30 a.m., her bag got ruptured, we took her to the delivery room and laid her on the table. Then she started having convulsions. So, immediately we have this injection of magnesium sulphate and it was administered. She was immediately referred. Now, it takes at least 30–45 minutes for the attendants, ambulance, and all the stuff to be arranged. She was taken to the district, and she was checked on the table. Though her fit was controlled from here itself, she was not in her senses. From the District Hospital she was being referred to the medical college, and when she was put on the stretcher, she expired*.’ (IDI, SN).

Women felt that respectful care without any abuse was the basic entitlement that they deserved from a health provider. One of the key parameters of good care in a health facility is that they should be taken care of and not shouted at or beaten.

*‘We do not go to hospital with the expectation of someone attending to or guiding us… all we want is for them not to use abusive language or scold us in the hospital and they should take good care and make efforts to keep the mother and the baby safe.’* (FGD, Women).

‘*They should take good care. If the patient is weak, they will scold her and expect her to cooperate fully and make effort by herself, but she has limited strength and will push only as much as she can. If women do not have strength how will they push beyond their strength? Even they should do something to ease their pain like give medicine or injection so that the delivery happens quickly without any difficulty. This is the reason why most women want to deliver in hospitals because there they expect to get these medicines and injections for speedy and less painful delivery procedure. At home such things are not available*.’ (FGD, Women).

Providers, while realising that respectful behaviour with patients was imperative, narrated situations when it was difficult for them to hold their temper back. Providers complained of the non-cooperative attitude of women and their family members, which often led to unpleasant interactions.

*‘There are many instances when I am compelled to lose my patience. Some patients talk to me rudely, some demand for facilities beyond our reach and some patients or family members want their patient to be given services and care on immediate basis which is sometimes not possible due to high patient load. But despite these instances I have to control my temper. I try to listen to them patiently and try my best to sort out their problems.’* (IDI, SN).

#### Client-provider communication and adequate information sharing: common understanding of the importance of sharing information of women’s condition, but women complain of lack of sharing of her condition with family, and providers cite pressure and difficult working conditions

From the women’s perspective, another key component that made for a positive patient-provider interaction was that providers shared information with the women about their condition, procedures required, and advice on care. Women reported apathy by providers in sharing such information with them.

*‘No, they didn’t say anything about these things. They didn’t inform us regarding newborn care at home; what to eat, etc.’* (FGD, Women).

The providers also acknowledged the need for sympathetic communication and adequate sharing of information.

*‘Providing adequate information about the procedures and giving appropriate clinical care are important factors that I give more priority to while providing obstetric and family planning care. One has to maintain good interpersonal relation…we have to show sympathy to them and talk to them. We have to lay them on the table and keep talking to them, comforting [them] while we take their vital signs, etc.’* (IDI, SN).

Providers mentioned that in spite of high patient load, they tried to share information pertaining to women’s condition, procedures to be performed, precautions to be taken and advice on postnatal care in the local language. Information is mostly shared with family members, particularly if there is any complication and referral is needed.

*‘Yes, before starting any procedure, I usually talk to the women about their present condition and what is expected out of them. I always communicate with women in local dialect so that they understand the information as it is conveyed. If I come to know that the woman is not being able to respond properly due to her condition, then I talk to the family members about her complication. Informing the relatives about the patient’s condition and what is expected out of them helps them to get a clear idea of the situation and relieves them of anxiety and stress.’* (IDI, SN).

#### Cost of tests and medicines from outside, and demand for informal payments by staff: financial burden on women but providers are helpless

Though entitled to free delivery services in public health facilities, women often had to spend on tests and medicines from outside, or in making informal payments to staff after delivery. These costs imposed a considerable financial burden on the women, most of whom belonged to families with low income.

Women and their families were asked to purchase medicines or get tests done from outside, which imposed considerable financial burden on them.

*‘If they wish they will come and see you once or give injection. When we request, they will say we don’t have the injection and ask us to get it from outside. If you complain, they don’t listen to you. People think that if you have come to deliver in a government facility you will not have to spend anything but this is not true*.’ (FGD, Women).

Informal payments to facility staff was highlighted in the FDGs and women reported having to make payments to facility staff following delivery.

*‘It’s like the nurse, midwife, and all these people, they ask for money even if there’s a stillbirth. Rather, it is the responsibility of the government to give us money.’* (FGD, Women).

Informal and unanticipated costs have negated the intended financial benefit from the JSSK incentive.

*‘As we have to get medicines and all these things from outside, we usually end up spending 1000 to 1200 rupees for that. We end up spending more than we actually receive as JSY money.’* (FGD, Women).

Providers, while realising the financial burden of additional costs imposed on the women for drugs and supplies, felt helpless as supplies, or the absence of crucial testing facilities, was not in their hands. They felt that at a minimum, supplies of essential drugs should be ensured by the District office to enable deliveries free of cost for the users.

‘*Now, there is shortage of oxytocin injections for many days. There is shortage of iron tablets for many days. If there are iron tablets, then it would help poor patients as they have to purchase costly syrups from outside*.’ (IDI, LMO).

#### No expectation of care at public health facilities: disillusionment among some women

Women in all focus groups voiced a certain disillusionment with public health facilities, either on account of poor past experience of care or upon hearing stories of other women’s poor experiences of delivery care. Such accounts made the women sceptical of expecting any improvement in the delivery care at public facilities.

*‘First of all, we don’t get facilities easily there. It doesn’t matter what facilities we wish to have there. What is there to expect if you don’t get any facilities there?’* (FGD, Women).

A few women, specifically those pregnant for the first time, expressed that they did not know what to expect in an institutional delivery and therefore had no expectations.

*‘We don’t know anything so what can we say! We can’t say anything about the facilities until we go there. What should we say now? Whatever facilities are available we are fine with it!*’ (FGD, Women).

The findings highlighted the contracting views of both women and providers and challenges that the providers has cited in providing care (Table 1)

**Table 1.** Perspectives of pregnant women and providers.

Themes of care	Women’s perspective	Provider’s Perspective	Challenges
**Structure of Care**			
Human resource	Delivery to be conducted by doctors and they should be available in the facilities.	Trained nurses are capable of conducting deliveries and doctors are available when needed.	Shortage of female doctors, especially non -availability at night due to lack of security and amenities in facilities.
Physical resource (medicine and supplies)	Medicine to be available free of cost and whenever needed.	Women and families demand medicines, often those that are not available at the facility. Management of complications is not possible due to inadequate supplies.	Stock outs of medicine and delay in replenishing from the district and lack of basic life saving supplies to manage complications.
Physical resource (food)	Food should be available for women and also their attendants during entire duration of stay.	Food is an essential requirement for delivering women and her attendants.	Lack of control over quantity and quality of food supplied due to district-appointed caterers.
Physical resource (ambulance service)	Functioning and readily available vehicle to reach the facility during emergency.	Ambulances could also serve as mobile labour rooms, particularly during emergencies.	Non- availability of driver during night.
**Process of care**			
Internationally recognised good practice (Cleanliness and Hygiene)	Overall cleanliness of facility, particularly clean toilets and bed sheets.	Overall hygiene and maintenance of cleanliness in the facility.	Lack of support staff and their contractual employment status lead to disruption of services.
Privacy	Need for screens between beds and delivery tables.	Curtain and separators between delivery tables and restricted entry of males in the examination and labour room.	Overcrowding leads to males entering restricted areas; lack of space in labour rooms and PNC wards leads to non-use of screens.
Good care’ – safe and respectful delivery with no complications	Prompt care and constant monitoring, with respect and dignity	Timely management of complications to ensure that both the mother and baby are healthy and safe.	Inability to manage complications due to lack of requisite infrastructure. Non-cooperative attitude of women and their family members towards providers
Cognition, (Client Provider interaction)	Sharing of information about their condition, any procedures if required, and advice on care.	Sympathetic communication and adequate sharing of information in local language.	Workload and time pressure
Financial cost of care	Free medicines and all tests needed during delivery.	Purchase of medicine and conducting tests outside facilities leads to financial burden on the women.	Lack of supplies and absence of necessary testing facilities.
No expectation of care	Previous poor experience, information from peers and lack of knowledge of entitlement, particularly for prima cases.		

## Discussion

The study identified themes of care from women’s perspectives, which were found to be equally relevant for the providers. A meaningful assessment of quality of care is possible only when it amalgamates the provision of care with the user experience of care [20]. We aimed to achieve this amalgamation by comparing and contrasting both provider and user perspectives of the quality of care received by them during institutional delivery [Table 2]. Among the ten elements of care constituting the Hulton framework, human and physical resources feature twice – as elements of both provision and experience of care. Our findings corroborate this, as both women and providers prioritised the availability of doctors and medicines at the facility and food for women and attendants. Referral services, an element of provision of care under the Hulton framework, also emerged in our study, equally prioritised by women and providers. This shows the importance attached by women to being able to reach the facility on time for delivery, or for treatment of a complication.

**Table 2.** Comparison of study themes with the Hulton’s framework.

Themes of Care as per Hulton Framework		Themes emerging in the study findings
Elements of both Provision and Experience of Care	Human and physical resources	Availability of doctors at the facility; availability of medicines and supplies free of cost; availability of food for women & attendants
Elements of provision of care	Referral system	Ambulance services prioritised by both women and providers
	Maternity information systems	–
	Use of appropriate technologies	‘Good care’- effective medicines and procedures
	Internationally recognised good practice	‘Good care’ – effective medicines and procedures; maintenance of cleanliness & hygiene
	Management of emergencies	‘Good care’ – safe delivery with no complications
Elements of experience of care	Cognition	Information sharing by provider
	Respect, dignity & equity	Privacy; ‘Good care’ in terms of promptness, respectful behaviour; no demand for informal payments
	Emotional support	–

Two elements – maternity information systems and emotional support – did not figure in the concerns voiced by women and providers. The former is an element of provision of care but evidently was not prioritised by providers. The latter is an element of the experience of care, but possibly women related emotional support to family and not so much the facility, and therefore did not have any specific expectations from facilities. However, their expectation of respectful behaviour, constant attention and cognitive care could be assumed to also include empathy and supportive care.

Among structural elements that emerged in this study, infrastructure, and availability of medicine, supplies, food, ambulance and trained providers was prioritised by both women and providers, have also emerged as important determinants of good care in other studies in similar settings [25–27]. However, there was a divergence in perspectives of women and providers with respect to delivery attendance, as women prefer to be delivered by a doctor. This was not relevant from the providers’ perspective, as trained nurses can handle deliveries. Similarly, there is a divergence in women’s and provider’s understanding of ‘good care’, which is to be expected. The former rate the care as per their understanding of what is ‘good’ or appropriate, while providers would rate good care as clinically appropriate care as per standard international protocols [28] Both providers and women cited lack of reliable transport as a major barrier in accessing care during delivery, like other study settings in LMICs [29–31]. Not only provision of reliable transportation systems, but also provision of trained paramedics in case of emergency situations plays a crucial role where accessibility is an issue in several countries [32–34].

In this study availability of food for the women and accompanying family members was regarded as an essential pre-requisite for overall satisfaction and sustained utilisation of maternity care at public health facilities. In terms of the quality of meals served in similar context, women often have expressed dissatisfaction over the quality received in a facility which can be a significant barrier for poor households [35–40].

The themes relating to process of care emerging in this study, including privacy, expectation of good care and safe delivery with no complications, maintaining hygiene and cleanliness, sharing of information, prompt care, constant attention, maintaining privacy and respectful care are all universally recognised themes of quality maternity care in similar study settings [27,37,41–48].

Privacy during institutional delivery, including antenatal and postnatal check-ups was ranked highly by women as well as providers, particularly ‘being shielded’ from other labouring women [27,45,49–51]. Space constrained labour-rooms and obstructions caused by the use of curtain in attending to multiple women simultaneously have been cited as potential reasons that providers do not use curtains for privacy [11,52–54]. Facility cleanliness in terms of clean linen in the wards, with frequent cleaning of the wards and toilets was a significant determinant of women’s overall satisfaction of services, which often are irregular and disorganised [55–59]. Management of complications as necessary, in addition to good health of the mother and child, are the most desirable aspect of care expressed by the women in this study as well as others,, as these elements affect their health and survival [27,37,41]. Similarly, inadequate information sharing with women and her family, disrespectful treatment by staff and poor provider attitude, are perceived to equate with poor quality by women in our study and others, and can be a deterrent for further seeking care in health facilities [43–46,60–66].

The cost of care, which was linked to availability of medicine and supplies in the facilities, was voiced by both women and providers as a concern as it often led to out of pocket expenditure, imposing an undue financial burden on the women in this study. Several studies have highlighted that the out of pocket expenditure for institutional delivery is higher than the monetary incentive provided by the government in many settings [67–69].

## Conclusion

This study has highlighted the themes of care that women value and want when they deliver in health facilities. This clearly points to the areas developing countries need to prioritise in improving the quality of maternity services – more doctors, all medicines available in the facility, better referral systems, more hygiene, respectful behaviour, better communication and more supportive care. Health systems in developing countries continue to focus on physical and human resources, augmenting supplies and infrastructure. But challenges remain, such as the absenteeism of doctors from rural facilities on account of difficult living and working conditions [70,71]. Augmenting drug supplies is also essential to prevent out of pocket expenditure. Sensitising all facility staff on respectful behaviour, therapeutic communication and supportive care is a critical element and needs to be incorporated as a compulsory training for all cadres. However, changing staff attitudes towards patients, particularly women from rural poor backgrounds, will take time. Enhancing provider skills and adopting international care protocols to ensure safe delivery needs to be a constant focus of the health system. There is also a need to inform and educate community women on good clinical care – the effort has to be towards minimising the misconceptions regarding medicines, tests and procedures among women. Adequate information sharing and communication by providers is one way by which women and families can be made to understand the care being imparted to them.

Mapping both user and provider perspectives for focused attention in quality improvement efforts can result in improved satisfaction among women seeking care, as well as the practitioners responsible for providing care.

## Supplementary Material

Supplemental Material

## Paper context

Increased institutional delivery in India has not adequately reduced maternal mortality, pointing at quality gaps. We explored women’s expectations of what should constitute as good-quality care in health facilities, and how much they match when compared with provider perspectives. Women highlighted the desire of care that is respectful, free of cost and ensures their basic entitlement in terms of trained personnel, medicine and food are provided. Most themes corroborated with provider’s perspectives on quality childbirth care. Understanding both perspectives and aligning with facility improvement interventions can lead to provision of high-quality care and thereby impacting maternal outcomes.
