# Supplementary material for: Do women’s perspectives of quality of care during childbirth match with those of providers? A qualitative study in Uttar Pradesh, India
Source: Glob Health Action. 2018 Oct 8;11(1):1527971. doi: 10.1080/16549716.2018.1527971 (PMC6179056; doi:10.1080/16549716.2018.1527971)
Supplement: Supplemental Material [file ZGHA_A_1527971_SM6187.zip › Appendix 1 .docx]

**Appendix 1: Profile of FGD participants (women)**

| **Characteristics of respondents** | | **Respondents (N=36)** |
| --- | --- | --- |
| Age | 19-22 | 10 |
|  | 23-26 | 19 |
|  | 27 and above | 7 |
| Education | Illiterate/no formal schooling | 14 |
|  | Primary Level (5 years) | 8 |
|  | Secondary (10 years) | 11 |
|  | Sr. Secondary above | 3 |
| Religion | Hindu | 32 |
|  | Muslim | 4 |
| Caste | Other Backward Caste | 25 |
|  | Scheduled Caste | 7 |
|  | Not disclosed | 4 |
| Occupation of respondents | Home maker | 35 |
|  | Tailor | 1 |
| Occupation of husbands | Agricultural labour | 4 |
|  | Non-agricultural casual labour | 15 |
|  | Self-employed outside agriculture | 10 |
|  | Salaried Employee | 3 |
|  | Un-employed | 3 |
| Number of ANC check-up | First | 1 |
|  | Second | 11 |
|  | Third | 24 |
| Place of ANC check-up | Government facility | 33 |
|  | Private facility | 1 |
| Parity | First | 10 |
|  | Second | 22 |
|  | Third or more | 4 |
| Place of last delivery | Government facility | 16 |
|  | Private facility | 6 |
|  | Home | 4 |
|  | Not applicable | 10 |
