# Supplementary material for: Do women’s perspectives of quality of care during childbirth match with those of providers? A qualitative study in Uttar Pradesh, India
Source: Glob Health Action. 2018 Oct 8;11(1):1527971. doi: 10.1080/16549716.2018.1527971 (PMC6179056; doi:10.1080/16549716.2018.1527971)
Supplement: Supplemental Material [file ZGHA_A_1527971_SM6187.zip › Appendix 2.docx]

**Appendix 2: Profile of IDI participants (providers)**

|  |  | **Medical Officer In charge (N=9)** | **Lady Medical Officer (N=9)** | **Staff Nurse (N=9)** |
| --- | --- | --- | --- | --- |
| Age (years) | 20-30 |  | 2 | 3 |
|  | 31-40 | 5 | 3 | 5 |
|  | 41-50 | 4 | 3 | - |
|  | 50+ |  | 1 | 1 |
| Qualification | MS  MBBS  BAMS | -  9  - | 1  6  2 |  |
|  | M.Sc. Nursing  Diploma in GNM  Sr. Secondary (12 years) |  |  | 2  6  1 |
| Previous Position/designation | MOIC  Medical Officer | 7  2 |  |  |
|  | LMO  Private Practitioner  None |  | 2  4  3 |  |
|  | Staff Nurse  None |  |  | 3  6 |
| Duration of service in current position | Less than 1 year | 4 | 2 | - |
|  | 1 -2 year | 2 | 3 | 3 |
|  | 3 or more | 3 | 4 | 6 |
| Years of total experience in Health Sector | Below 5 years | 1 | 4 | 6 |
|  | 5 years or more | 8 | 5 | 3 |

Acronyms - MS Master of Surgery; MD Doctor of Medicine; MBBS Bachelor of Medicine Bachelor of Surgery; BAMS Bachelor of Ayurvedic Medicine and Surgery; GNM Diploma in General Nursing and Midwifery; B.Sc. Nursing Bachelor of Science in Nursing
